# CRISPR-tagging mice in aging research

**DOI:** 10.18632/aging.101566

**Published:** 2018-09-19

**Authors:** Joseph M. Miano, Xiaochun Long

**Affiliations:** 1 Aab Cardiovascular Research Institute, University of Rochester Medical Center, Rochester, NY 14642, USA; 2 Department of Molecular and Cellular Physiology, Albany Medical College, Albany, NY 12208, USA

**Keywords:** CRISPR, epitope tagging, mouse, antibody

Aging is an independent risk factor for cardiovascular, neoplastic, and neurodegenerative diseases. Several genes have emerged as important regulators of signal transduction pathways and gene programs linked to longevity, including members of the *Sirtuin* (*SIRT*) [1] and *FOXO* [2] gene families and *KLOTHO* (*KL*) [3]. The accurate detection, localization, and binding-association of these and other gene-encoded proteins (and their splice variants) are of paramount importance in advancing an understanding of age-related processes. However, antibodies often are not rigorously tested for specificity and papers get published with questionable data. For example, the predicted molecular weight of the protein encoded by *KL* is 116 kDa. Yet, Western blotting studies in vascular cells report smaller molecular weights of KL without demonstrating specificity of the bands with, for example, siRNA knockdown experiments [3]. Commercial resources of antibodies similarly often fail to report specificity measures to ensure accurate detection of a protein. The result of such lack in experimental rigor can be perpetuation of false data over the course of many years, even when the published data are in accord with the predicted molecular weight of the protein. This was precisely the case with a cardiovascular transcriptional coactivator known as Myocardin (MYOCD) [4], the estimated molecular weight of which is 100 kDa. For over 16 years, scores of papers reported MYOCD with a molecular weight of ~100 kDa as well as dubious localization using unauthenticated antibodies [5]. Even more frustrating was the lack of success from many labs in generating reliable antibodies to MYOCD. It seemed that a discriminating antibody for endogenous MYOCD was without reach.

Meanwhile, new advances in genome editing were evolving, most notably the revolutionary clustered regularly interspaced short palindromic repeats (CRISPR)/CRISPR-associated protein 9 (Cas9) system [6]. This technology involves a user-defined single guide RNA (sgRNA) that shepherds Cas9 (both available from Synthego, www.synthego.com) to a specific sequence in the genome where a protospacer adjacent motif (PAM) exists. Upon contacting the PAM and contiguous complementary sequence recognized by the sgRNA, Cas9 creates a DNA double-strand break that undergoes rapid mending by the cell’s endogenous repair machinery. In the presence of a repair template, typically a single-strand oligonucleotide (ssODN), precise sequences – even single base substitutions – become incorporated into the newly repaired segment of the genome. This astonishing technology has been a huge game-changer for biomedical research, to say nothing of the therapeutic potential [6]. Accurately detecting MYOCD, or other problematic proteins of virtually any species, could now be solved easily by knocking in a small epitope tag with CRISPR. This was done in mice and the results revealed MYOCD protein to be of molecular weight ~150 kDa, much larger than the predicted (and reported) molecular weight of ~100 kDa [7]. Mass spectrometry proved the presence of MYOCD peptides at this molecular weight. Further proteomic studies will likely reveal post-translational modifications of MYOCD that account for the higher-than-predicted molecular weight. Further studies revealed expected nuclear localization in cultured vascular smooth muscle cells and binding activity over specific DNA sequences near known target genes using a ChIP assay in aorta [7]. Since both alleles of *Myocd* are transcriptionally active [7], it should be possible to obtain mice with each *Myocd* allele CRISPR-tagged with a unique peptide (eg, 3xFLAG and 3xHA). This offers further experimental rigor in detecting MYOCD whether in a cell or bound to other macromolecules.

Only about 1% of the CRISPR literature pertains to aging. What are some important considerations and how might CRISPR-tagging of proteins assist in further elucidating the biology of aging? Before CRISPR-tagging a gene locus in the mouse, investigators should determine what tag to incorporate. If, for example, eGFP is desired for live cell imaging, then a larger repair template will be required (eg, Megamer from Integrated DNA Technologies). It should be noted that unless a cell type is impossible or difficult to culture in vitro, CRISPR-tagging mice is preferred over cell lines since a mouse model affords both in vivo and in vitro experimentation and the time to generate such a mouse is remarkably fast (~5-6 weeks). Another important factor in CRISPR-tagging is ascertaining whether the tag should be placed at the amino or carboxy terminus of a protein. For example, tagging the carboxy terminus will often capture all splice variants of a protein. Preliminary experiments would be necessary to ensure protein activity and localization are not altered with the position of the tag. Apart from demonstrating the accurate molecular weight and true cellular localization of a longevity protein, CRISPR-tagging with 3xFLAG or 3xHA will allow for rigorous ChIP-seq of highly related proteins such as those of the FOXO family [2]. By using different tags for each FOXO paralog, specific target genes may be disclosed under varying experimental conditions. Pulldown experiments could also be done to define new binding partners of a protein by mass spectrometry. This might require a high affinity tag such as biotin. RNA immunoprecipitation followed by next generation sequencing (RIP-seq) is yet another application of CRISPR-tagging a protein. RIP-seq allows for the detection of the ever- expanding class of long non-coding RNAs (lncRNAs) that have increasingly varied and complex modes of action in various biological processes. Indeed, there are numerous lncRNAs implicated in senescence and age-related diseases, but there is limited knowledge as to the interacting proteins [8]. It is likely that novel lncRNAs associate with key proteins in a ribonucleoprotein complex that impacts signaling and gene expression changes associated with senescence and aging processes. Finally, CRISPR-tagged mice could undergo subsequent rounds of genome editing of putative enhancers or post-translationally-modified amino acids to track any consequent changes in expression, localization, protein-protein association or activity.
